# METTL21A, a Non-Histone Methyltransferase, Is Dispensable for Spermatogenesis and Male Fertility in Mice

**DOI:** 10.3390/ijms23041942

**Published:** 2022-02-09

**Authors:** Jinmei Li, Shenglei Feng, Xixiang Ma, Shuiqiao Yuan, Xiaoli Wang

**Affiliations:** 1 Institute of Reproductive Health, Tongji Medical College, Huazhong University of Science and Technology, Wuhan 430030, China; jinmeili@hust.edu.cn (J.L.); 2018512006@hust.edu.cn (S.F.); mxx52yx@163.com (X.M.); 2 Laboratory Animal Center, Huazhong University of Science and Technology, Wuhan 430030, China; 3 Research Institute of Huazhong University of Science and Technology in Shenzhen, Shenzhen 518057, China

**Keywords:** mettl21a, methyltransferases, spermatogenesis, fertility, meiosis

## Abstract

Protein methyltransferases play various physiological and pathological roles through methylating histone and non-histone targets. Many histone methyltransferases have been reported to regulate the development of spermatogenic cells. However, the specific function of non-histone methyltransferases during spermatogenesis remains unclear. In this study, we found that METTL21A, a non-histone methyltransferase, is highly expressed in mouse testes. In order to elucidate the role of METTL21A in spermatogenesis, we generated a Mettl21a global knockout mouse model using CRISPR/Cas9 technology. Unexpectedly, our results showed that knockout males are fertile without apparent defects in the processes of male germ cell development, including spermatogonial differentiation, meiosis, and sperm maturation. Furthermore, the ablation of METTL21A does not affect the expression and localization of its known targeting proteins in testes. Together, our data demonstrated that METTL21A is not essential for mouse spermatogenesis and male fertility.

## 1. Introduction

Spermatogenesis is an extremely complex developmental process and involves the orderly differentiation of multiple types of spermatogenic cells, including mitotically proliferating spermatogonial cells, meiotically dividing spermatocytes, and spermatids that eventually mature into spermatozoa [1]. In recent years, an increasing number of studies focused on the critical roles of epigenetic regulation in spermatogenesis [2]. Protein methylation, a post-translational modification, has been revealed to regulate a wide array of cellular functions ranging from RNA metabolism to chromatin structure remodeling, DNA repair, gene transcription, protein synthesis, and signal transduction [3].

With advances in mass spectrometry and characterization of the methylproteome, numerous protein methyltransferases (PMTs) were identified, which catalyze the methylation of histones and non-histone proteins [4]. Interestingly, many histone methyltransferases have been demonstrated their ability to regulate the development of germ cells at different stages of spermatogenesis. For instance, histone methyltransferase ESET can regulate the apoptosis of spermatogonial stem cells (SSC) by suppressing *Cox4i2* expression by histone H3 lysine 9 tri-methylation (H3K9me3) [5]; combined depletion of EZH1 and EZH2, which are two protein methyltransferase that cause a depletion of global H3K27me3 marks and meiotic arrest in spermatocytes [6]; and conditional ablation of SETD2, a key methyltransferase catalyzing the trimethylation of histone H3 lysine 36 (H3K36me3), results in aberrant spermiogenesis with acrosomal malformation, leading to complete infertility [7].

Meanwhile, many methylated non-histone proteins and the enzymes that introduce them govern various biological processes [4], but their roles in spermatogenesis remain unclear. In humans, a group of methyltransferase-like 21 (METTL21) proteins (A–E) has recently been identified to mediate the lysine methylation of molecular chaperones and eukaryotic translation elongation factor 1A and closely relate with human health and disease [3,8,9,10,11,12]. Specifically, METTL21A catalyzes the methylation of HSP70 at Lys-561, which has been strongly linked to cancer and is often upregulated in tumors [13]; METTL21B could target Lys-165 in eEF1A in a GTP-dependent manner and has been described as one of the epigenetic factors for multiple sclerosis [9]; METTL21C, a protein-lysine N-methyltransferase catalyzing the Lys315 trimethylation in valosin-containing protein (VCP), can regulate calcium homeostasis in muscles and promote the differentiation of myoblasts to myotubes [10,11]. METTL21D, another VCP-methyltransferase, can promote tumor metastasis by affecting cell growth, migration, and infection [11,14]. METTL21E was previously demonstrated to maintain myofiber size by inhibiting proteasome-mediated protein degradation [12]. Among these METTL21 methyltransferases, METTL21A is the member with the most known target proteins reported so far [13]. Interestingly, these targets of METTL21A are known as heat shock proteins (HSPs), including HSP70, GRP75, GRP78, and HSC70, which have been associated with the pathogenesis of male infertility [15,16,17,18]. However, the knowledge about the function of METTL21A on spermatogenesis is largely unknown.

In this study, to investigate the role of METTL21A in male germ cell development and spermatogenesis, we first detected the expression of METTL21A in various mouse tissues. We found that it was predominantly expressed in testes, thus prompting us to establish *Mettl21a* knockout mice to explore its physiological function in spermatogenesis. Unexpectedly, our data revealed that *Mettl21a* knockout mice were completely fertile without any defects in the processes of germ cell development, including spermatogonial differentiation, meiosis, and sperm maturation. Furthermore, we found that *Mettl21a* ablation does not influence the expression level and subcellular localization of its target proteins.

## 2. Results

### 2.1. Mettl21a Is Preferentially Expressed in Mouse Testes

**Figure 1:**
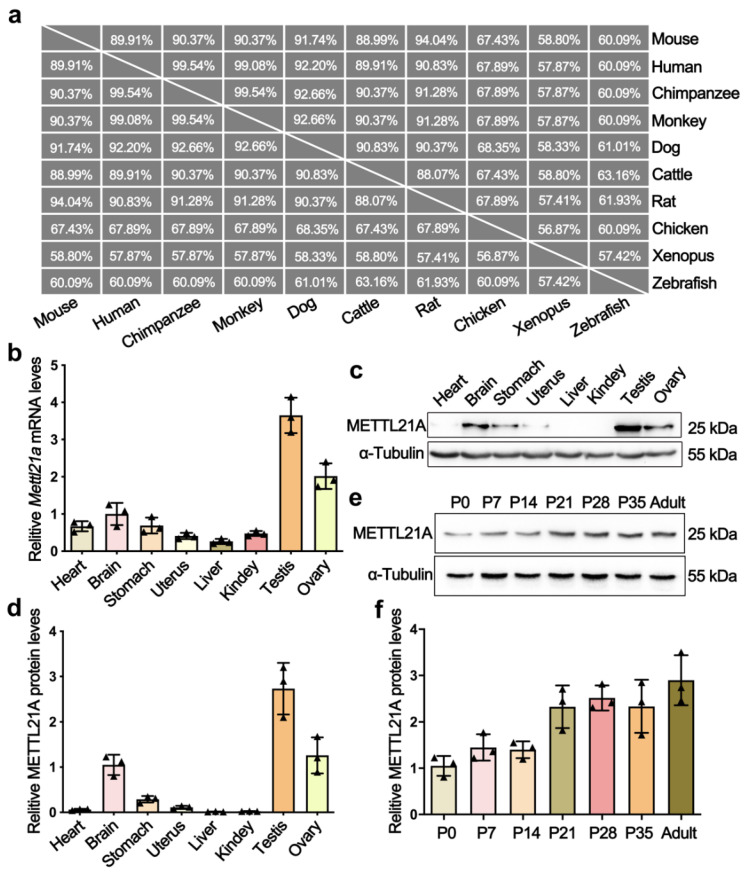
The expression profiles of METTL21A in multiple tissues and developing testes from mice. (**a**) A high degree of conservation of METTL21A in amino acid sequences was acquired from the NCBI database among 10 species. (**b**) RT-qPCR showing *Mettl21a* mRNA levels in multiple organs using *Arbp* as internal control. Data are presented as mean ± SEM, *n* = 3. The three values are indicated as three triangles on each bar. The following bar charts are the same unless otherwise stated. (**c**) Western blot is showing the protein levels of *Mettl21a* in various organs. (**d**) Quantification of relative *Mettl21a* protein levels in (**c**). (**e**) Western blot showing the protein levels of *Mettl21a* in developing testes. (**f**) Quantification of relative *Mettl21a* protein levels in (**d**). Data are presented as mean ± SEM, *n* = 3.

Phylogenetic analyses and multi-alignment of METTL21A orthologs in 10 vertebrate species revealed that METTL21A is highly conserved during evolution (Figure 1a). To explore the expression profile of *Mettl21a*, we examined its mRNA and protein levels in multiple tissues from adult mice by RT-qPCR and Western blot analyses, respectively. The results showed that both mRNA and protein levels of *Mettl21a* are predominantly expressed in testis, followed by ovary, brain, and stomach (Figure 1b–d). We further investigated the spatial expression of *Mettl21a* during spermatogenesis and found that *Mettl21a* is continuously expressed from postnatal day 0 (P0) until P56 and displays the highest level at P56 (Figure 1e,f). These findings suggest a potential role of METTL21A in the regulation of spermatogenesis.

### 2.2. Generation of Mettl21a Knockout Mice

**Figure 2:**
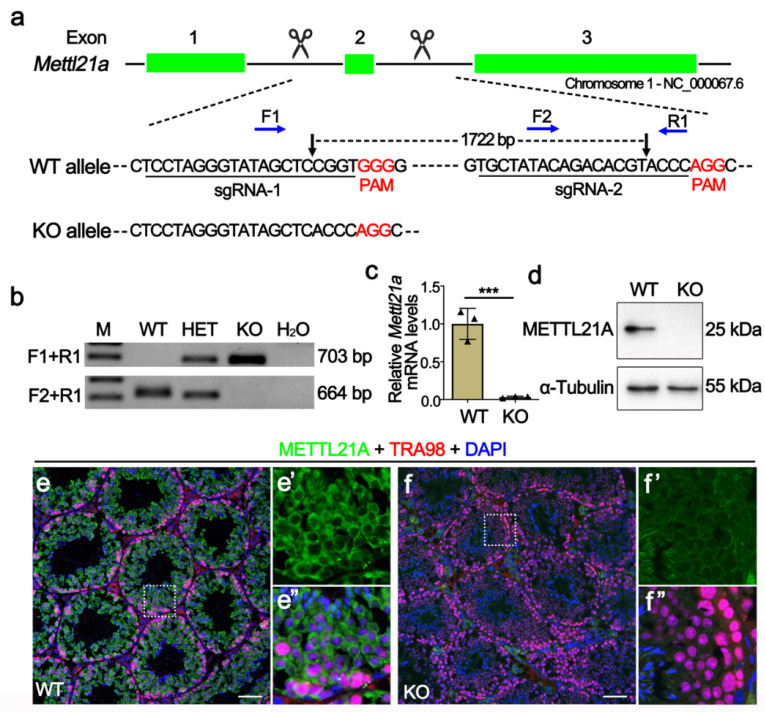
Generation of *Mettl21a* knockout mice using CRISPR/Cas9 technology. (**a**) Schematic representation of the targeting strategy for generating *Mettl21a* knockout (KO) mice using the CRISPR/Cas9 system. Green boxes represent exons of the *Mettl21a* gene on mouse chromosome 1; black underlines indicate the target regions of sgRNAs and black arrows show the cut sites, blue arrows indicate the sites of forward (F) and reverse (R) primers for genotyping. (**b**) Representative PCR genotyping results of *Mettl21a* alleles show that the WT allele is a longer band (703bp) using F1 and R1 primers, and the mutant allele is a shorter band (664bp) using F1 and R2 primers. M, marker; Het, heterozygous; H_2_O, negative control. (**c**) RT-qPCR analyses of *Mettl21a* mRNA levels in P56 WT and KO testes. Data are presented as mean ± SEM, *n* = 3. *** *p* < 0.001 by student’s *t*-test. (**d**) Western blot showing the METTL21A protein levels in WT and KO testes at P56. α-Tubulin served as a loading control. (**e**–**f’’**) Co-immunofluorescence staining of METTL21A and TRA98 (a nuclear marker of pan-germ cells) in WT and KO mouse testicular sections. Nuclei were stained with DAPI. Scale bars = 50 µm. Magnified images (**e’**,**e’’**,**f’**,**f’’**) of white boxes in (**e**) and (**f**), further highlight the changes in METTL21A localization and expression between WT and KO testes.

In order to elucidate the physiological role of METTL21A in spermatogenesis, we created *Mettl21a* knockout mouse model using CRISPR/cas9 technology. Two sgRNAs were, respectively, designed to target introns flanking the exon 2 (Figure 2a), thus generating a 1722-bp deletion verified by Sanger sequencing analysis, which resulted in frame-shift mutations of the *Mettl21a* gene. Specific primers for wild-type (WT) or homozygous mutant (KO) alleles were designed for genotyping (Figure 2b). RT-qPCR and Western blot confirmed the absence of both mRNA and protein of *Mettl21a* in KO testes (Figure 2c,d). Furthermore, by co-staining METTL21A and TRA98 (a nuclear marker of pan-germ cells), we found that METTL21A is mainly localized in the cytoplasm of germ cells, but it is absent in the KO testes (Figure 2e–f’’). Taken together, our data demonstrate that the *Mettl21a* gene was successfully knocked out in mice.

### 2.3. Mettl21a Is Dispensable for Spermatogenesis

**Figure 3:**
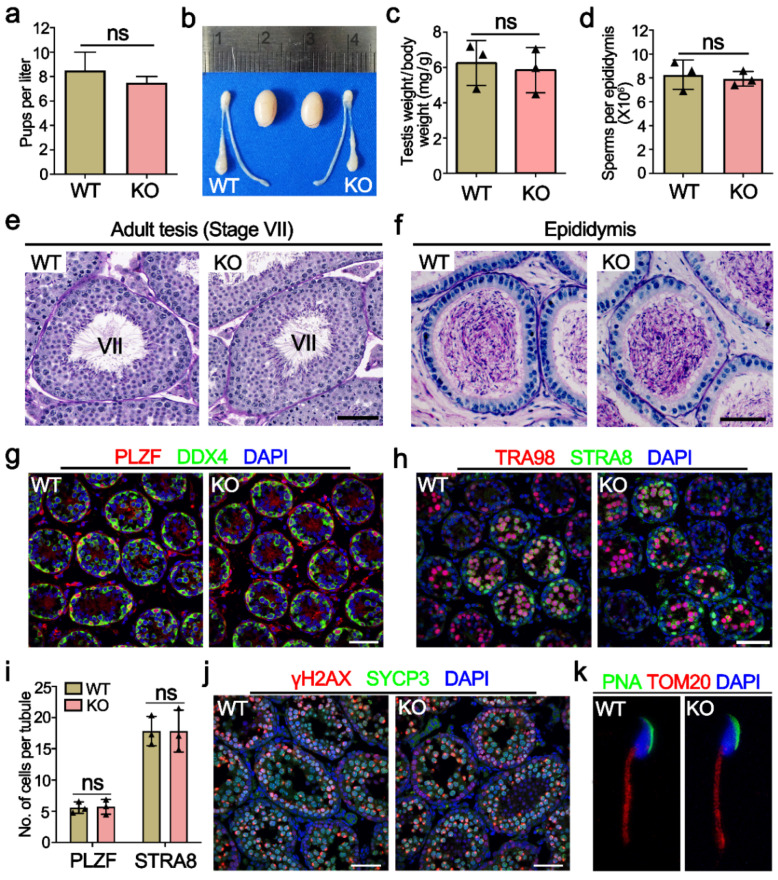
METTL21A is dispensable for spermatogenesis in mice. (**a**) The number of pups per litter derived from WT and KO males at the ages of 6–12 weeks. Data are presented as mean ± SEM, *n* = 8. ns, not significant. (**b**) The gross morphology of the testes from P56 WT and KO mice. (**c**) The ratio of testis weight to the body weight of 8-week-old WT and KO mice. Data are presented as mean ± SEM, *n* = 3. ns, not significant. (**d**) The sperm concentration of 8-week-old WT and KO mice. Data are presented as mean ± SEM, *n* = 3. ns, not significant. (**e**,**f**) Periodic acid-Schiff (PAS) staining of testes (**e**) and cauda epididymides (**f**) paraffin sections from 8-week-old WT and KO mice. Scale bar = 50 μm. (**g**) Co-immunofluorescence staining of PLZF and DDX4 in P10 WT and KO mouse testes. Scale bar = 50 µm. (**h**) Co-immunofluorescence staining of TRA98 and STRA8 in P10 WT and KO mouse testes. Scale bar = 50 µm. (**i**) The quantification of PLZF^+^ and STRA8^+^ cells per tubule at P10 in WT and KO testes, respectively. Data are presented as mean ± SEM, *n* = 3. ns, not significant. (**j**) Co-immunofluorescence staining of γH2AX and SYCP3 in P21 WT and KO testes. Scale bars = 50 µm. (**k**) Co-immunofluorescence staining of PNA and TOMM20 in spermatozoon from P56 WT and KO cauda epididymis.

*Mettl21a* KO mice were viable and developed normally, and fertility test by interbreeding adult WT or KO males with WT females for six months yielded no significant change in the number of litters and the average number of offspring per litter between WT and KO males **(**Figure 3a), indicating that *Mettl21a* KO males are completely fertile. Furthermore, adult *Mettl21a* KO males were indistinguishable from their WT littermates for testis size, testis/body weight ratio, the epididymal sperm count, and histological appearance of seminiferous tubule sections (Figure 3b–e). Seminiferous tubules from *Mettl21a* KO males harbor a full array of spermatogenic cells, including spermatogonia, spermatocytes, round spermatids, and elongated spermatids (Figure 3e). Consistent with these findings, a comparable number of mature spermatozoa was observed in the lumen of epididymis from WT and *Mettl21a* KO males (Figure 3f). These data suggest that spermatogenesis was not grossly impaired in the absence of *Mettl21a* in mice.

In order to address more subtle defects, we immunostained several markers of germ cells and found no significant difference between WT and *Mettl21a* KO testis, including spermatogonia makers PLZF (undifferentiated spermatogonia maker) and STRA8 (a marker of differentiating spermatogonia and pre-leptotene spermatocytes) (Figure 3g–i); spermatocyte marker SYCP3 (a component of the axial elements of the synaptonemal complex); and DSB marker γH2AX (a phosphorylated form of the histone variant H2AX that accumulates upon formation of DSBs) (Figure 3j). Furthermore, *Mettl21a* KO spermatozoa display normal morphology labeled with acrosome marker PNA and mitochondrial marker TOM20 (labeling the midpiece of spermatozoa) (Figure 3k).

### 2.4. Deletion of Mettl21a Did Not Alter the Expression of Its Target Proteins and Other Mettl21 Members

**Figure 4:**
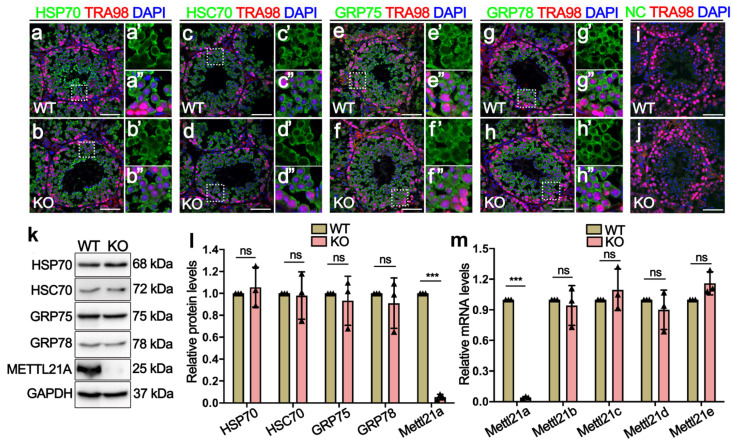
*Mettl21a* deletion does not affect the temporal and spatial expression of its target proteins. (**a**–**h’’**) Co-immunofluorescence staining of TRA98 and HSP70 (**a**,**b**), HSC70 (**c**,**d**), GRP75 (**e**,**f**), and GRP78 (**g**,**h**), respectively, in P56 WT and KO testes. Scale bar = 50 µm. Magnified images (**a’**,**a’’**,**b’**,**b’’**,**c’**,**c’’**,**d’**,**d’’**,**e’**,**e’’**,**f’**,**f’’**,**g’**,**g’’**,**h’**,**h’’**) of white boxes in (**a**–**h**), further highlight the expression and localization of HSP70, HSC70, GRP75, and GRP78 in WT and KO testes, respectively. (**i**,**j**) Negative control (NC) omitting the primary antibody to detect the specificity of the immunostaining with Alexa 488. Germ cells show unspecific staining. Germ cell were stained with anti-TRA98 antibody. Nuclei were stained with DAPI. Scale bars = 50 µm. (**k**) Western blot showing the expression of HSP70, HSC70, GRP75, GRP78, and METTL21A in P56 WT and KO testes. GAPDH was used as a loading control. (**l**) Quantification of relative indicated protein levels of B, respectively. Data are presented as mean ± SEM, *n* = 3. ns, not significant. *** *p* < 0.001 by student’s *t*-test. (**m**) RT-qPCR shows the mRNA levels of five members of the *Mettl21* family in adult WT and KO testes. Data are presented as mean ± SEM, *n* = 3. ns, not significant. ***p < 0.001 by student’s *t*-test.

Considering that *Mettl21a* can affect the expression and localization of its target proteins (HSP70, HSC70, GRP75, and GRP78) through methylation modification [19], we performed IF and Western blot assay to determine the localization and protein levels of its target proteins in *Mettl21a* KO testes. Similarly to the cellular distribution of METTL21A, these target proteins are also mainly located in the cytoplasm of germ cells and exhibit no apparent changes in *Mettl21a* KO testes compared with WT testes (Figure 4a–j). In addition, their protein levels are also indistinguishable between WT and *Mettl21a* KO testes (Figure 4k–l).

In order to clarify whether a compensatory regulation of *Mettl21* family members occurs in *Mettl21a* KO males, we quantified testis mRNA levels of a panel of *Mettl21* protein family by RT-qPCR, including *Mettl21a, Mettl21b, and Mettl21c*; *Mettl21d;* and *Mettl21e*. The results showed no significant changes among those Mettl21 family genes in *Mettl21a* KO testes compared with WT testes (Figure 4m). These findings suggest that the loss of *Mettl21a* was not sufficient to affect spermatogenesis in mice.

## 3. Discussions

Increasing evidence has revealed the pivotal role of epigenetic factors in the pathophysiology of male infertility. With rapid advancements in epigenetic research, the importance of protein methylation has been highlighted. Approximately 200 methyltransferases were predicted to be encoded by the human genome, although most remain uncharacterized concerning the targeted substrates and the functional roles [20]. Given the critical biological functions and implications of methyltransferases in human diseases, there has been a growing interest in assessing these enzymes as potential therapeutic targets [4]. Considering the key function of METTL21A in catalyzing the methylation of multiple heat shock factors that have been associated with the pathogenesis of male infertility [16], we explored whether it plays a role in spermatogenesis. Interestingly, although *Mettl21a* was found to be highly expressed in mouse testis, *Mettl21a* knockout male mice were completely fertile without any apparent defects.

Methylation of nonhistone proteins is supposed to modify their stability, activity, and molecular interactions [21]. Previous studies revealed that METTL21A could catalyze methylation of Lys561 in human HSP70 protein, resulting in the translocation of HSP70 from the cytoplasm to the nucleus, thereby linking HSP70-K561me2 to cell proliferation via stimulating the kinase activity of Aurora kinase [19]. However, in this study, the ablation of METTL21A did not influence the subcellular localization and protein levels of its targets in seminiferous tubules. Due to the lack of specific antibodies against these methylated targets, we were unable to obtain a more accurate analysis for methylation levels of these proteins, but we cannot rule out the following possibilities: (1) METTL21A does not methylate HSP70 and other known targets in mouse germ cells; (2) if these target proteins can be catalyzed by METTL21A in mouse testes, their methylation modifications do not affect their stability and subcellular localization; (3) when METTL21A is deleted, other protein methyltransferases might compensate its absence, although we did not observe a significant increase in the mRNA level of the other METLL21 homologs. Therefore, we conclude that METTL21A is dispensable for mouse spermatogenesis, despite whether it mediates the methylation of HSP70 or not in male germ cells.

To date, over 2300 testis-enriched genes have been identified, which have long been considered essential genes regulating spermatogenesis [22]. However, previous studies have established a number of KO mouse models and found that many testis-enriched genes are dispensable for male fertility [23,24]. Similarly, we identified *Mettl21a*, which was mainly enriched in the testes and was not required for spermatogenesis and fertility in mice. To our knowledge, this is the first study to explore the in vivo function of *Mettl21a*. Our findings will help prevent other researchers in the field of reproductive genetics from conducting redundant experiments and serve as a fundamental resource for genetics studies on human fertility.

## 4. Materials and Methods

### 4.1. Animals and Ethics Statement

All animal procedures were approved by the Institutional Animal Care and Use Committee (IACUC) of Tongji Medical College, Huazhong University of Science and Technology, China. The mice were housed in the specific pathogen-free facility of Laboratory Animal Center in Huazhong University of Science and Technology. All experiments with mice were conducted ethically according to the Guide for the Care and Use of Laboratory Animal guidelines.

### 4.2. CRISPR-Cas9 Mediated Gene Targeting and Genotyping

For *Mettl21a* knockout mouse generation, sgRNAs were designed targeting the introns flanking the exon 2. In vitro transcribed guide RNA and Cas9 (T7 Transcription Kit from Ambion, Austin, TX, USA) were mixed and microinjected into pronuclei of CBA/J × C57BL/6J hybrid zygotes and transferred into the oviducts of pseudopregnant females. *Mettl21a* mutant founder mice were created and crossed with wild-type (WT) mice to obtain heterozygous mice. Then, heterozygous mice carrying1722-bp deletion of *Mettl21a* allele were inbred to produce homozygous mice (KO). The sequences of sgRNA are 5′-TCCTAGGGTATAGCTCCGGT-3′ and 5′-TGCTATACAGACACGTACCC-3′, respectively. Genotyping was performed by PCR amplification of genomic DNA extracted from mouse tails. The PCR primers for the *Mettl21a* mutant allele were Forward: 5′- CTTAGTTAGCTGCCCAGTGAGG-3′; and Reverse: 5′-AACAAATGGAACAAACGAGGTGTC-3′, yielding a 703 bp fragment. PCR primers for *Mettl21a* wild-type allele were Forward: 5′-CTTGATTAGAGAGGCAGAGATGG-3′; and Reverse: 5′-AACAAATGGAACAAACGAGGTGTC-3′, yielding a 664 bp fragment.

### 4.3. Fertility Test

Each male mouse (6–12 weeks, *n* = 5) was continuously caged with two WT C57BL/6 females (6–12 weeks), which were checked for vaginal plugs every morning. Once a vaginal plug was identified, another female was placed in the cage for another round of mating. The plugged female was separated and singly caged, and the pregnancy was recorded, and the number of pups was counted after birth. The fertility test lasted for 4 months.

### 4.4. Tissue Collection and Histological Analyses

Testes and epididymis from at least three mice for each genotype were collected when the mice were killed after cervical dislocation and fixed in Bouin’s solution (Sigma, HT10132) at 4 °C overnight. Then, the samples were dehydrated stepwise by using an ethanol series (70%, 90%, and 100% ethanol), embedded in paraffin, and cut into 5 µm tissue slices. After dewaxing and hydration, the sections were stained with periodic acid-Schiff (PAS) and imaged with a FluoView 1000 microscope (Olympus, Tokyo, Japan) with a digital camera (MSX2, Micro-shot Technology Limited, Guangzhou, China).

### 4.5. Sperm Counting

The cauda epididymis was dissected from indicated genotypes of mice at P56. Then, sperm was squeezed out from the cauda epididymis and incubated in HTF medium for 30 min at 37 °C under 5% CO_2_. Sperm counting analyses were performed according to previous literature [25].

### 4.6. Total RNA Extraction and RT-qPCR

Total RNAs were extracted from the indicated samples using TRIzol reagent (Invitrogen, Waltham, MA, USA) and treated with DNase I (Dnase-free kit, Invitrogen) to remove residual genomic DNA. Then, the RNA (2.5 µg) from each sample was reverse-transcribed into cDNA using the PrimeScript RT Reagent Kit (Takara, Shiga, Japan) according to the manufacturer’s protocol. RT-qPCR was performed with SYBR green master mix (TaKaRa) on LightCycler@96 Real-Time PCR system (Roche, Basel, Switzerland), and specific forward and reverse primers sequences were listed in Supplementary Table S1. Relative gene expression was analyzed using the 2^−ΔΔCt^ method, and housekeeping gene *Arbp* was used as an internal control. This method was used for analyzing data in Figure 1b, Figure 2c, and Figure 4m.

### 4.7. Immunostaining

Testes were fixed in 4% paraformaldehyde (PFA) in PBS at 4 °C overnight. The samples were dehydrated stepwise through a series of sucrose solutions (5%, 12.5%, and 20%), embedded in OCT, and cut into 5 µm tissue slices. Then slides were boiled in 0.01 M sodium citrate buffer (pH = 6.0) for 15 min using a microwave for antigen retrieval. After blocking, the sections were incubated with primary antibodies (Supplementary Table S2) overnight at 4 °C and with the secondary antibodies for 1 h at room temperature. The images were taken by FluoView 1000 microscope (Olympus, Tokyo, Japan) with a digital camera (MSX2, Micro-shot Technology Limited, Guangzhou, China).

### 4.8. Protein Extract and Western Blot

The proteins from indicated samples were extracted using RIPA lysis buffer (CWBIO, Cat# 01408). After being denatured with 5 × SDS loading buffer (Beyotime, P0015L) at 100 °C for 10 min, the protein lysates were separated in 10% SDS–PAGE gels and transferred onto PVDF membranes (Bio-Rad). The membranes were blocked with 5% skimmed milk for 1 h at room temperature and then incubated at 4 °C overnight with the primary antibodies and with secondary antibodies (Supplementary Table S2) at room temperature for 1 h. Subsequently, the Luminol/enhancer solution and Peroxide solution (ClarityTM Western ECL Substrate, Bio-Rad) was used for photographs using the ChemiDoc XRS+ system (Bio-Rad).

### 4.9. Statistical Analysis

All data are presented as mean ± SEM unless otherwise noted in the figure legends. Statistical differences between datasets were assessed by Student’s *t*-test using the SPSS16.0 software. *p*-values are denoted in figures by *** *p* < 0.001.

## Data Availability

Not applicable.

## Supplementary Materials

The following supporting information can be downloaded at: https://www.mdpi.com/article/10.3390/ijms23041942/s1.
